# The New Horizon: A Viewpoint of Novel Drugs, Biomarkers, Artificial Intelligence, and Self-Management in Improving Kidney Transplant Outcomes

**DOI:** 10.3390/jcm14145077

**Published:** 2025-07-17

**Authors:** Artur Quintiliano, Andrew J. Bentall

**Affiliations:** 1 Division of Nephrology and Hypertension, Mayo Clinic, Rochester, MN 55905, USA; dasilva.artur@mayo.edu; 2 Department of Medicine, Federal University of Rio Grande do Norte, Natal, RN 59078-970, Brazil; 3 The William J von Liebig Center for Transplantation and Clinical Regeneration, Mayo Clinic, Rochester, MN 55905, USA

**Keywords:** kidney transplantation, chronic kidney disease, biomarkers, artificial intelligence, patient self-management, drug, novel therapeutics

## Abstract

The increasing prevalence of chronic kidney disease (CKD) and end-stage kidney disease (ESKD) has led to a growing demand for kidney transplantation (KTx). Identifying risk factors that enable improved allograft survival through novel therapeutic agents, advanced biomarkers, and artificial intelligence (AI)-driven data integration are critical to addressing this challenge. Drugs, such as SGLT2 inhibitors and finerenone, have demonstrated improved outcomes in patients but lack comprehensive long-term evidence in KTx patients. The use of biomarkers, including circulating cytokines and transcriptomics, coupled with AI, could enhance early detection and personalized treatment strategies. Addressing patient self-management and addressing health access disparities may be more achievable using technologies used at home rather than traditional models of healthcare and thus lead to increased transplant success, both in terms of transplantation rates and allograft longevity.

## 1. Introduction

The increasing prevalence of chronic kidney disease (CKD) and end-stage kidney disease (ESKD) in recent years has led to a growing demand for renal replacement therapy (RRT) [1]. The current RRT requirements are estimated at 2436 per million population (pmp) in the United States (USA—2021), 1187 pmp in Europe (2021) [2], and 758 pmp in Brazil (2022) [3]. For comparison, the prevalence of RRT in the USA in 2009 was 1700 pmp, marking a 43.3% increase over 12 years [4]. In particular, kidney transplantation (KTx), due to its improved survival rates and quality of life benefits, has seen an increasing demand [5]. However, health systems worldwide have struggled to meet this increasing need [6], as organ availability has remained relatively static. Specifically, the number of deceased donor kidney transplants in the USA rose from 8654 in 2003 to 18,691 in 2021. This corresponds to an increase in kidney donation rates from 54 to 102 per 1000 potential donors [7]. During the same period, the number of kidney transplant recipients in the United States increased only modestly, from 16,487 in 2012 to 26,309 in 2022 [8,9]. Improving post-transplant management, including the prediction of graft survival, is crucial in healthcare systems in order to reduce the organ allocation to patients who return to the already-burdened transplant waiting list. This paper aims to explore potential advancements in renal allograft survival through the introduction of new drugs into the care regimen for KTx, associated risk assessment tools, new and traditional biomarker integration with electronic health record (EHR) data using artificial intelligence (AI) analysis, and using AI to enhance patient self-management.

To ensure methodological rigor and clarity, the authors applied the Scale for the Assessment of Narrative Review Articles (SANRA) framework [10], which strengthens the scientific validity of narrative reviews. The significance of this topic lies in the need to assess the impact of emerging strategies and technologies on kidney graft survival, particularly in light of rapid advancements in pharmacological therapies, biomarker integration, and AI-driven decision-making in transplant medicine. A comprehensive literature search was conducted using PubMed, focusing on English-language journals and prioritizing peer-reviewed articles published within the last 10 years. The selection process emphasized high-quality research that contributes to a deeper understanding of long-term transplant outcomes. A key relevant endpoint explored in this review is long-term allograft survival, as it remains a critical challenge in the field. By integrating these elements, this review aims to provide a structured and evidence-based analysis of how novel interventions may enhance kidney transplant longevity and patient outcomes.

## 2. Innovative Pharmacological Strategies in Kidney Transplantation

Sodium-glucose transport protein 2 inhibitors (SGLT2is) have emerged as a new class of therapeutics with beneficial effects on both cardiovascular (CV) and kidney outcomes in patients with CKD, irrespective of the presence of diabetes or proteinuria. In this population, SGLT2i have demonstrated benefits in slowing CKD progression and heart failure (HF) events [11]. Glycemic control, whilst beneficial, does not seem the main mechanistic pathway for improved outcomes and so this is an additional therapeutic option to renin–angiotensin–aldosterone blockade agents (angiotensin-converting enzyme inhibitors (ACEi)/angiotensin II receptor blockers (ARBs)), but not validated in KTx recipients. Given the additional mechanisms of action with SGLT2i, they could be beneficial in KTx recipients with diabetes mellitus (DM), glomerular lesions, or HF to improve allograft longevity and cardiovascular risk [12], addressing the unmet needs of improving longer-term outcomes.

Kidney transplant recipients may have several factors that could limit the efficacy of SGLT2i therapy or expose patients to unwarranted risk, specifically related to their immunosuppressed state and urinary tract infection risk. Thus, currently, all published large, randomized controlled trials examining the safety and efficacy of SGLT2i have excluded KTx recipients [13,14]. Consequently, there is a lack of well-designed trial evidence for the use of SGLT2 inhibitors in KTx despite their unique therapeutic promise in this population [15,16,17] and limited data on the long-term outcomes of SGLT2i therapy in KTx, including overall and CV-related mortality or allograft survival. The existing evidence in the published literature addressing KTx focuses only on short-term outcomes and varies significantly in study design, population characteristics, duration of follow-up, and measured outcomes, making it difficult to compare studies or draw meaningful conclusions [18,19,20]. Additionally, proteinuria and mesangial expansion, hallmarks of diabetic nephropathy, are present in transplant glomerulopathy and long-term biopsy studies [21,22].

Another promising treatment for CKD, finerenone, a non-steroidal mineralocorticoid receptor antagonist (MRA), has potential for KTx patients. The FIDELIO-DKD study demonstrated improvement in proteinuria in diabetic kidney disease with reduced eGFR decline (by 18%) and less renal failure and cardiovascular events over 2.6 years of follow-up [23]. Given the burden in KTx recipients, this could be useful in post-transplant proteinuria; however, the interaction with calcineurin inhibitors (CNIs) and hyperkalemia would require increased surveillance, particularly with polypharmacy, and is currently under investigation [24]. The widespread efficacy in more diverse KTx populations is also required, given the differing responses to therapies in, for example, Black and Hispanic populations, considering the impact of both genetic and psychosocial aspects of care [25,26]. Thus, the potential future combination of these two classes of medications in KTx patients may also enhance graft longevity.

New immunosuppression agents in KTx, avoiding the side effects of CNI (cyclosporin or tacrolimus), are being trialed, including sipilizumab and tegoprubart, to avoid the nephrotoxicity associated with chronic CNI use [27]. The role of the tolerance and avoidance of immunosuppression has been shown to be beneficial [28,29].

## 3. Causes of Allograft Failure

Alloimmune causes of transplant failure are still the most common cause of death-censored graft failure [30,31]. Early allograft failure is more associated with surgical complications; however, after 6 months, the majority of histological lesions seen in for-cause biopsies are alloantibody-related [32]. Rejection within the first year is associated with allograft failure [33]; however, the reduction in one-year rejection rates to 7.9% [34] has not translated into the 40–50% of allografts lost from alloimmune causes, predominantly through chronic antibody-mediated rejection over time [35]. Progression in kidney allografts to failure is often a multifactorial process that involves immunological and non-immunological mechanisms of kidney injury [36]. Immune factors include acute rejection (AR), histocompatibility, and immunosuppressive therapy compliance, while nonimmune risk factors also play a role, including poor donor quality, ischemia–reperfusion injury (IRI), nephrotoxicity, hypertension, and diabetes, as well as infections [37]. Progressive interstitial fibrosis/tubular atrophy (IF/TA) is a significant pathological characteristic of allograft dysfunction [18], but it is often hard to specifically identify the underlying mechanisms [31,37,38,39]. HLA matching may play a role; however, despite HLA identical siblings, chronic histological lesions still occur [40,41]. Furthermore, more detailed tissue typing matching has been associated with de novo HLA donor-specific antibody (DSA) reduction; however, the impact on longer-term allograft function has not been described [41,42,43].

## 4. Biomarkers

Biomarkers are used to evaluate the biological process, pathogenic process, or pharmacological response to a therapeutic intervention [44] and can be classified according to Table 1 [45]. There are many pathophysiological changes in KTx involving both the donor and recipient. Donor changes can occur prior to procurement, but IRI takes place with transfer, and reperfusion is an independent risk factor for delayed graft function (DGF) [46]. Markers for assessing donor quality include biomarkers such as neutrophil gelatinase-associated lipocalin (NGAL) associated with ischemic kidney injury [47]; kidney injury molecule-1 (KIM-1), a marker of early kidney proximal tubular cell injury [48], and urinary N-acetyl-β-D-glucosaminidase (uNAG) is also a marker of proximal tubular injury [49].

The commonly targeted settings for the use of biomarkers in KTx are IRI, DGF, and cellular and antibody-mediated rejection, and these biomarkers rely on structural changes in the kidney, detectable immunological changes, or circulating donor DNA in the recipient [38].

New biomarkers for early and late graft dysfunction are essential in clinical practice to improve the management of complications and prolong graft survival in KTx. A wide range of potential diagnostic and prognostic biomarkers, measured in different biological fluids and in renal tissues, have been proposed for DGF and chronic allograft dysfunction (CAD). However, traditional biomarkers, such as eGFR, proteinuria, donor-specific antibodies, Doppler ultrasound, histological parameters, and others, and new potential biomarkers (circulating inflammatory cytokines KIM-1, Tumor necrosis factor receptor 1—TNFR-1, Tumor necrosis factor receptor 2—TNFR-2, monocyte chemoattractant protein-1—MCP-1, soluble receptor of urokinase plasminogen activator—suPAR, and YKL-40) have their limits and strengths [38]. More recently, mortality has been associated with cytokine changes, particularly growth differentiation factor-15 (GDF-15) and interleukin-6 (IL-6) [50]. Summary details on these new biomarkers can be found in Table 2.

OMICs technology has allowed identifying many candidate biomarkers, providing diagnostic and prognostic information at very early stages of pathological processes, such as AR. OMICs refers to a set of high-throughput techniques used to analyze the complete set of molecules within a biological system, providing a comprehensive understanding of its structure, function, and dynamics at different molecular levels. These technologies focus on various biological domains, including genomics, transcriptomics, proteomics, metabolomics, and epigenomics [51].

Several promising tools are emerging, including donor-derived cell-free DNA (ddcfDNA) [54], extracellular vesicles (Evs), and components of the innate immune system, such as complement activation in IRI, where donor urinary C5a levels have been independently associated with post-transplant DGF in recipients [52]. Additionally, potential biomarkers for antibody-mediated rejection (ABMR) and urine albumin-to-creatinine ratio ACR [55,56] using urine and peripheral blood transcriptomics are also promising tools [57].

Although most of these biomarkers require validation in multiple independent cohorts and standardization, they may pave the way for significant advancements, whether as individual assay use or potentially in combination to increase the yield in either sensitivity to the outcome or biological relevance to the individual patient. These include the ability to accurately predict the risk of DGF before allograft implantation, detecting subclinical rejection at a molecular level before histological lesions develop, and identifying the underlying causes of chronic allograft dysfunction. Furthermore, the identification of patients with immunological tolerance allows for the minimization of immunosuppressive therapy, which represents another active area of research [38]. Non-invasive imaging may play a role in chronic progressive allograft dysfunction [58].

## 5. Artificial Intelligence Integration into Healthcare

Precision medicine is increasingly pivotal in KTx, offering a proactive approach to predicting and preventing pathological processes by providing earlier and more comprehensive insights than traditional methods [55]. The concurrent advancements in biomarker research and AI play a crucial role in harnessing the vast array of biomarker data. Prospective studies are essential to determine whether integrating these novel biomarker sets into clinical practice—via AI-assisted risk model development—could enhance long-term graft survival compared to current standards, thereby addressing gaps in the existing literature.

A practical example of the integration of biomarkers and clinical data is KidneyIntelX, a predictive model that combines known biomarkers with EHR data to forecast longitudinal kidney outcomes. This model has been applied to two high-risk patient populations, which include patients with type 2 Diabetes Mellitus (T2DM), and an African American registry database of patients with the APOL1 risk allele. The composite renal outcome was rapid decline in kidney function (GFR of ≥5 mL/min/1.73 m^2^/year), a 40% sustained GFR decline, or ESKD within 5 years. The AUC for KidneyIntelX was 0.77 in T2DM and 0.8 in patients with APOL-1 risk alleles, representing meaningful prediction for outcomes [59].

The early identification of high-risk patients for progression to ESRD is crucial for the optimal allocation of limited healthcare resources and the implementation or intensification of evidence-based interventions to slow kidney function decline. However, predicting kidney disease progression in clinical practice remains challenging, particularly in patients with largely preserved kidney function [60]. Two primary challenges contribute to the difficulty of early identification and prediction. The most used biomarkers (eGFR and UACR) are relatively insensitive and nonspecific, exhibiting significant fluctuations and variability in the early stages of CKD [61], but perform consistently well in the prediction of progression. Furthermore, the prevailing approach relies on predictive models that incorporate only a single baseline measurement of a selected feature without accounting for longitudinal data, thereby limiting their predictive accuracy [62]. Integrating these biomarkers with dynamic clinical data through machine learning techniques has demonstrated significant potential to enhance the discrimination and prediction of composite kidney endpoints [59].

This integrated approach has immediate clinical implications, particularly when incorporated into clinical decision support systems (CDSSs) and embedded within EHR. For instance, patients with a high KidneyIntelX risk score, indicating a probability exceeding 50% for adverse kidney outcomes, should be referred to a nephrologist—a measure that has been associated with improved clinical outcomes [63]. Alternatively, patients with a low-risk KidneyIntelX score may be appropriately managed by their primary care provider through standard-of-care treatment with the routine monitoring of their KidneyIntelX results. In contrast, patients with an intermediate-risk score should undergo standard care with periodic retesting. Over time, these patients may exhibit changes in their KidneyIntelX scores influenced by behavioral modifications, evolving clinical parameters, and treatment adjustments, necessitating appropriate clinical interventions as required. This stratified approach not only enhances individualized patient management but also optimizes healthcare resource utilization by addressing the uncertainty surrounding the referral of patients to a limited number of subspecialists. This has been demonstrated in improved diabetes care using an AI model of derived healthcare delivery, with improved outcomes compared to traditional office visit healthcare [64]. Consequently, it contributes to the more efficient allocation of specialized medical services and improves overall healthcare system efficiency [59].

## 6. Artificial Intelligence

Classical statistical models, such as the Kaplan–Meier estimator, logistic regression, and Cox proportional hazards models, generally assume the independence of predictors and primarily focus on survival estimation by identifying the most influential variables. However, these models are not inherently designed to account for complex interactions among predictors and are often inadequate for modeling non-linear relationships between predictors and outcomes [65]. Furthermore, the assumptions underlying statistical modeling—such as linearity, normality, and equality of variance—pose additional challenges. These assumptions limit the applicability of classical models, particularly when dealing with a large number of predictors, rendering them less effective in high-dimensional datasets [66].

A predictive model for graft survival can serve as a valuable decision-making tool for nephrologists, facilitating therapeutic and counseling decisions for patients [67]. If an optimal predictive model suggests a high likelihood of graft failure within five years, clinicians may need to explore strategies to improve survival outcomes based on the patient’s specific profile and selected predictive factors. These strategies may include optimizing immunosuppressive regimens, recommending earlier biopsies, and assisting patients in making informed decisions regarding their post-transplant management. Managing multiple medical problems together may improve clinician decision-making, such as combining cancer risk with immunosuppression with metabolic and obesity risks and being dependent on the allograft GFR.

A systematic review of published predictive models utilizing AI techniques to forecast graft failure after KTx analyzed 18 studies conducted in the United States since 2010. The review identified artificial neural networks, decision trees, and Bayesian belief networks as the most employed AI methods. The findings indicated that, based on reported improvements in predictive performance, AI holds significant potential to enhance KTx outcome prediction and support clinical decision-making [68].

**Figure 1:**
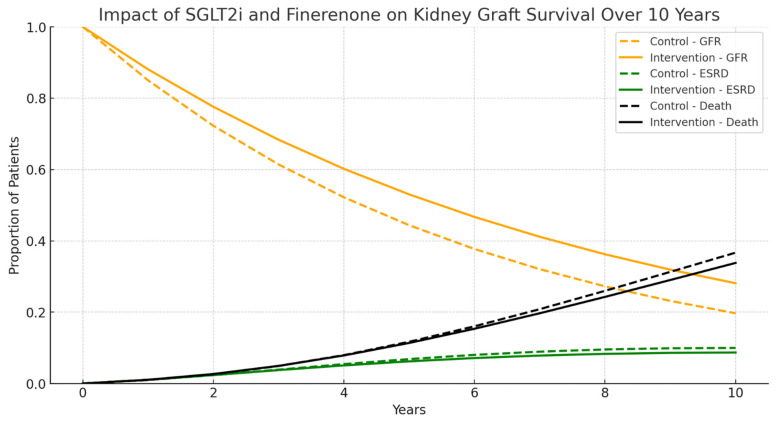
Impact of SGLT2i and finerenone simulation on kidney graft survival over 10 years.

Despite the proliferation of predictive models for renal outcomes utilizing AI, there is a scarcity of data incorporating models that include simultaneously new drugs used in the treatment of CKD, such as SGLT2i and finerenone, especially since they have not yet been introduced in KTx patients, biomarkers, and the potential for patient self-management in disease care, particularly in addressing health inequities. The Markov model [69] is widely used to simulate the progression of chronic diseases and the evolution of clinical states over time. We applied this model to kidney transplantation to assess the impact of an intervention with SGLT2 inhibitors and finerenone, simulating the trajectory of graft function and allograft failure under different scenarios—Figure 1. This simulation underscores the potential benefits of SGLT2 inhibitors and finerenone in preserving kidney graft function and reducing the risk of graft failure over a 10-year period. The findings suggest that integrating these medications into post-transplant management could achieve the following:

✔ Increase the proportion of patients maintaining stable kidney function by 42.8% (GFR > 60 mL/min).
✔ Reduce the progression to ESRD by 13%.
✔ Lower the incidence of mortality by 7.8%.

Figure 1 compares the progression of kidney function in transplanted patients under a traditional scenario (control group) versus a scenario where these medications are used (intervention group). Dashed lines represent the control group (without intervention), assuming the maintenance of the same results observed in the clinical trial population. Solid lines represent the intervention group (with SGLT2 inhibitors and finerenone).

The computational power available today has the potential to analyze vast amounts of data on interactions, enhancers, and attenuators, which earlier models were unable to predict with high accuracy and give clinicians better decision-making capacity. In addition, being able to model patient outcomes will help patients visualize the potential benefits of taking new medications compared to no change on an individualized platform. Studies that integrate these parameters could bridge critical gaps in our current understanding of renal disease management.

Using models with AI to allow patients to self-manage and adjust medications in a continuous feedback mechanism using virtual “bot” assistants would potentially improve outcomes using real-time continuous data sources [70]. For transplant outcomes, this would need to be proved to be safe and effective for multiple dependent variables, such as immunosuppression levels, viremia, and leukocyte counts. A similar model has performed well in diabetes and could be adapted for transplantation [64].

## 7. Self-Management

The contemporary era of CKD management is characterized by the integration of novel and promising pharmacological therapies, the emergence of biomarkers capable of prognosticating patient phenotypes predisposed to a rapid decline in renal function or graft loss, and the application of AI models capable of processing extensive datasets with refined precision. However, these advancements will not yield meaningful clinical outcomes unless patients actively engage in their treatment and may benefit from insight as displayed in Figure 1. This necessitates a commitment to lifestyle modifications, adherence to prescribed medication regimens, and the early identification of potential health complications.

Therefore, we have entered an era where novel and promising medications have become integral to the patient care continuum for CKD, new biomarkers have emerged, capable of prognosticating patient phenotypes that are predisposed to a rapid decline in renal function or graft loss, and AI models capable of extensive data processing with refined information. However, none of these advancements will yield meaningful outcomes unless patients take an active role in their treatment. This necessitates a commitment to lifestyle changes, adherence to regular medication regimens, and the early identification of health complications.

Patients with lower educational levels or socioeconomic status often encounter significant barriers that adversely affect their post-transplantation outcomes. Studies indicate that individuals lacking health insurance or a stable income source may experience difficulties in accessing healthcare services, including transplantation opportunities [71,72].

Educational level represents an important outcome determinant in KTx [73], as evidenced by a strong correlation between limited health literacy and reduced access to transplantation [74]. Individuals with higher socioeconomic and educational levels are more likely to pursue living donor transplantation [75]. Furthermore, several studies have identified educational level as a significant predictor of post-transplant outcomes, with lower levels being associated with an increased risk of graft failure, delayed graft function, patient mortality, and non-adherence to medication regimens [76]. Lower education levels are linked to higher risks of graft failure, delayed graft function, patient mortality, and non-adherence to medication regimens. However, limited research has explored the variability in post-transplant outcomes based on educational attainment and interventions to specifically improve this metric in transplant care [77]. Overcoming barriers in society to healthcare is an important public health issue, but an individual transplant center can seek to overcome local barriers to education in its transplant program, whether this relates to language, culture, or literacy.

However, improving patient outcomes is not solely dependent on overcoming structural barriers; it also requires comprehensive support for patient engagement and autonomy. In this regard, individuals with CKD benefit greatly from structured education and continuous interaction with healthcare providers. Physicians, nurses, and allied health professionals play a pivotal role in empowering patients by fostering trusting relationships, adapting communication to individual needs, and delivering personalized education strategies [78]. Effective support requires the development of relational and communication skills, which are particularly relevant in nephrology and dialysis settings, where patients face complex regimens and emotional stress [79]. Educational interventions tailored to health literacy, cultural background, and psychosocial context have shown positive impacts on adherence, quality of life, and clinical outcomes [80].

Therefore, policies aimed at mitigating health inequities—particularly among minority populations and individuals with lower educational levels and income—are needed [76,81]. These, alongside educational interventions designed to enhance patient self-management, especially in the early recognition of health deteriorations, have the potential to significantly improve mortality and graft survival outcomes in KTx patients. Moreover, these new data can be incorporated into predictive models, potentially integrating aggravation coefficients to mathematically identify vulnerable subgroups, even through propensity score matching within these analyses [82].

## 8. Conclusions

The incorporation of novel pharmacological therapies into the care regimen for KTx recipients, alongside increased patient awareness of the critical role of self-management, holds significant potential for improving transplant outcomes. Effective self-management empowers patients to take an active role in their healthcare, fostering better adherence to medication regimens, the implementation of lifestyle modifications, and the early detection of complications. Moreover, the integration of emerging biomarkers with traditional markers can facilitate the identification of subtle changes indicative of potential complications or graft rejection.

When these datasets are analyzed using advanced AI tools, the predictive accuracy of clinical outcomes can be enhanced. AI, coupled with rapid advancements in computational power, can process vast and complex datasets, uncovering patterns and correlations that might be overlooked by conventional analytical approaches—particularly those that fail to account for critical patient factors such as medication adherence and the ability to recognize early signs of complications. Consequently, AI-driven predictive models can enable the development of more precise, individualized treatment plans tailored to specific patient risks and needs.

**Figure 2:**
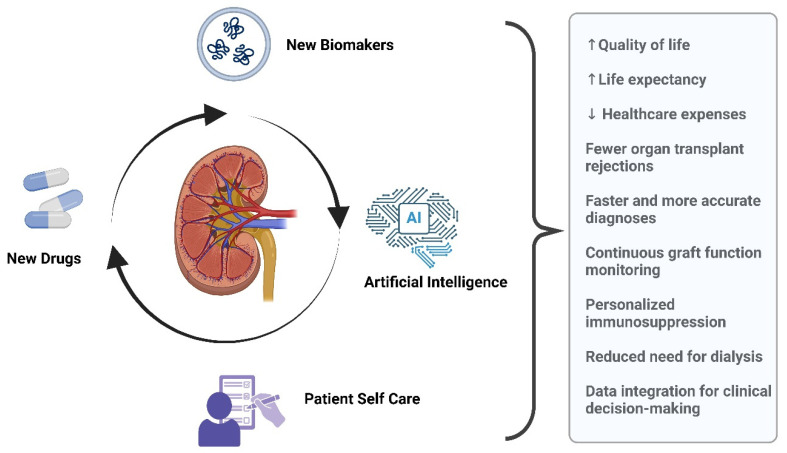
Approaches to improve kidney graft health. The integration of new biomarkers, artificial intelligence, novel drugs, and patient self-care creates a continuous cycle of improvement in kidney graft health.

Thus, the synergistic combination of novel pharmacological interventions, improved patient self-management, and the integration of advanced biomarkers analyzed through AI-driven methodologies represents a comprehensive and highly promising strategy for optimizing kidney transplant outcomes—Figure 2.

**Limitations and Strengths:** This narrative review provides a comprehensive and integrative synthesis of emerging strategies in kidney transplantation, offering a broad perspective on novel pharmacological therapies, biomarker integration, and AI-driven decision-making. By incorporating recent evidence from high-impact peer-reviewed studies, this review highlights cutting-edge advancements that have the potential to improve allograft survival and patient outcomes. Additionally, the discussion bridges scientific research with clinical applicability, emphasizing future directions and practical implications in transplant medicine.

However, as a narrative review, this work has certain limitations. The absence of a systematic methodology introduces a potential selection bias in the included literature, as there are no standardized inclusion or exclusion criteria. Furthermore, the lack of quantitative analysis or meta-analytic approaches prevents a statistical assessment of the impact of the discussed interventions. Despite these limitations, this review provides valuable insights into evolving post-transplant management strategies, synthesizing current knowledge to inform future research and clinical practice, and provides data to demonstrate the significant impact that interventions can have in improving transplant care.

## Figures and Tables

**Table 1:** Classification and examples of biomarkers in kidney transplantation.

Biomarker Category	Definition	Example(s)
Susceptibility or Risk Biomarker	Estimates the risk of developing a condition in a stable graft without clinical signs of dysfunction	Acute rejection (AR)
Diagnostic Biomarker	Identifies patients with a disease or a subset of it	AR type
Prognostic Biomarker	Estimates the likelihood of a clinical event or disease progression, staging disease severity	Severe rejection with risk of graft loss
Predictive Biomarker	Estimates the likelihood of achieving a favorable response to a therapy	Eculizumab for complement-fixing donor-specific antibodies (DSAs)
Monitoring Biomarker	Serially measured to detect disease evolution, drug toxicity, or exposure to immunosuppressive drugs	Tacrolimus levels
Pharmacodynamic/Response Biomarker	Verifies that a biological response has occurred following drug exposure	DSA mean fluorescence intensity (MFI) after ABMR treatment
Safety Biomarker	Estimates the presence and severity of drug-related toxicity	Calcineurin inhibitor nephrotoxicity

ABMR: antibody-mediated rejection; AR: acute rejection; DSAs: donor-specific antibodies; MFI: mean fluorescence intensity. Adapted from Naesens et al. (2018) [45].

**Table 2:** Biomarkers for acute kidney injury and kidney transplant outcomes: diagnostic and prognostic insights.

Biomarkers	Comment
Damage vs. stress biomarkers to predict AKI	Damage biomarkers had better predictive ability for AKI than the stress biomarker in various clinical settings.
NGAL/Cr urinary and serum/urinary NGAL	Serum NGAL and urinary NGAL are the most utilized biomarkers for AKI, both demonstrating high diagnostic accuracy regardless of whether they are adjusted for urinary creatinine. Among critically ill patients, various biomarkers exhibited comparable predictive performance for AKI. However, in non-critical patients, NGAL, NGAL/Cr, and serum NGAL showed superior diagnostic accuracy. In medical patients, NGAL demonstrated the highest diagnostic accuracy, whereas in surgical patients, NGAL/Cr was the most accurate biomarker for AKI diagnosis [47].
KIM-1	KIM-1 correlated with donor serum creatinine, while urinary KIM-1 was associated with delayed graft function [48].
OMICs	Encompassing different disciplines, such as the following: genomics (the study of the complete genome), transcriptomics (the analysis of gene transcripts—RNA), proteomics (the study of proteins expressed in a biological system), metabolomics (the analysis of metabolites present in cells and tissues), and epigenomics (the study of epigenetic modifications that regulate gene expression) [51].
Gene signatures	Gene signatures are specific sets of genes whose differential expression is associated with a particular biological condition, disease, or therapeutic response. For example, genes related to fibrosis, such as mRNA for vimentin, can be used to assess tissue remodeling and disease progression [38].
TLR-4 surface expression	It is reduced in DGF and associated with poor graft function at follow-up [38].
Non-HLA DSA	Pretransplant levels associated with acute and chronic antibody-mediated rejection, the severity of microvascular inflammation, graft dysfunction, and graft loss [38].
Fascin and Vimentin	Expression of these EndMT biomarkers on microvasculature correlated with long-term graft function after delayed graft function [38].
Mitochondrial DNA	It predicts DGF in donation after cardiac death donors [38].
Plasma and Urinary Endothelial EVs	EV levels and their procoagulant activity progressively decrease after KTx, paralleling renal function recovery [52].
Serum CXCL10	CXCL10 is involved in developing renal diseases through the chemoattraction of inflammatory cells and facilitating cell growth and angiostatic effects. Several studies have demonstrated that urinary CXCL10 expression is significantly elevated during AKI. The pretransplant elevation of serum CXCL10 concentration in patients with acute rejection shows an association with the risk of graft failure. Urinary CXCL10 levels increase in patients experiencing acute rejection [53].

AKI: acute kidney injury, CXCL10: C-X-C motif chemokine 10, DGF: delayed graft function, DSA: donor-specific antibodies, EndMT: endothelial to mesenchymal transition, EV: extracellular vesicles, HLA: human leukocyte antigen, KIM-1: kidney injury molecule-1, KTx: kidney transplant, NGAL: neutrophil gelatinase-associated lipocalin, OMIC: comprehensive study of sets of biomolecules in biological systems, and TLR-4: soluble toll-like receptor 4. Adapted from Quaglia et al. (2020) [38].

## Abbreviations

ABMR	antibody-mediated rejection
ACEis	angiotensin-converting enzyme inhibitors
ACR	acute T-cell-mediated rejection
AI	artificial intelligence
AR	acute rejection
ARBs	angiotensin II receptor blockers
CAD	chronic allograft dysfunction
CDSSs	clinical decision support systems
CKD	chronic kidney disease
CNIs	calcineurin inhibitors
CNPq	National Council for Scientific and Technological Development
CR	chronic rejection
CV	cardiovascular
ddcfDNA	donor-derived cell-free DNA
DGF	delayed graft function
DM	diabetes mellitus
DSAs	donor-specific antibodies
eGFR	estimated glomerular filtration rate
HERs	electronic health records
EndMT	endothelial to Mesenchymal Transition
ESKD	end-stage kidney disease
EV	extracellular vesicles
GDF-15	growth differentiation factor-15
HF	heart failure
IF/TA	interstitial fibrosis/tubular atrophy
IL-6	interleukin-6
IRI	ischemia–reperfusion injury
KIM-1	kidney injury molecule
KTx	kidney transplantation
MCP-1	monocyte chemoattractant protein-1
MFI	mean fluorescence intensity
MRA	mineralocorticoid receptor antagonist
NGAL	neutrophil gelatinase-associated lipocalin
Pmp	per million population
RRT	renal replacement therapy
SGLT2is	sodium-glucose transport 2 inhibitors
suPAR	soluble receptor of urokinase plasminogen activator
TLR-4	soluble Toll-like receptor 4
TNFR-1	Tumor necrosis factor receptor 1
TNFR-2	Tumor necrosis factor receptor 2
T2DM	type 2 Diabetes Mellitus
USA	United States of America
UACR	urine albumin-to-creatinine ratio
uNAG	urinary N-acetyl-β-D-glucosaminidase
